# *MYCN* gene polymorphisms and Wilms tumor susceptibility in Chinese children

**DOI:** 10.1002/jcla.22988

**Published:** 2019-07-25

**Authors:** Xiaokai Huang, Jie Zhao, Jinhong Zhu, Shanshan Chen, Wen Fu, Xiaoqian Tian, Susu Lou, Jichen Ruan, Jing He, Haixia Zhou

**Affiliations:** ^1^ Department of Hematology The Second Affiliated Hospital and Yuying Children's Hospital of Wenzhou Medical University Wenzhou China; ^2^ Department of Clinical Laboratory Biobank Harbin Medical University Cancer Hospital Harbin China; ^3^ Department of Pediatric Surgery, Guangzhou Institute of Pediatrics, Guangdong Provincial Key Laboratory of Research in Structural Birth Defect Disease, Guangzhou Women and Children's Medical Center Guangzhou Medical University Guangzhou China

**Keywords:** *MYCN*, polymorphism, susceptibility, Wilms tumor

## Abstract

**Background:**

Wilms tumor, derived from embryonic cells, accounts for a large proportion of pediatric renal tumors. MYCN encoded by *MYCN* proto‐oncogene, a member of the MYC family, is a BHLH transcription factor. It plays a critical role in tumorigenesis and predicts poor clinical outcomes in various types of cancer. However, the role of MYCN remained unclarified in Wilms tumor. In this study, we investigated the association between *MYCN* gene polymorphisms and Wilms tumor susceptibility.

**Methods:**

Four *MYCN* gene polymorphisms (rs57961569 G > A, rs9653226 T > C, rs13034994 A > G, and rs60226897 G > A) were genotyped in 183 cases and 603 controls. Adjusted odds ratios (AORs) and 95% confidence intervals (CIs) were calculated to evaluate the association between *MYCN* gene polymorphisms and Wilms tumor susceptibility.

**Results:**

Overall, no significant association was found for any of the four *MYCN* gene polymorphisms. Interestingly, in the stratification analysis, the rs57961569 was found to be associated with decreased Wilms tumor susceptibility in the children older than 18 months (AOR = 0.65, 95% CI = 0.42‐1.00, *P* = .050). Moreover, older children carrying 2‐4 risk genotypes were at increased risk of Wilms tumor (OR = 1.55, 95% CI = 1.001‐2.40, *P* = .0497). Haplotype GCAA was shown to significantly increased Wilms tumor risk (AOR = 2.40, 95% CI = 1.12‐5.14, *P* = .024).

**Conclusion:**

Our study demonstrated that these *MYCN* gene polymorphisms might be low penetrant variants in Wilms tumor.

## 1 INTRODUCTION

Wilms tumor is a classic embryonal tumor in the developing kidney, with a 90% cure rate.^1^, ^2^, ^3^ Despite the high cure rate in patients with low‐risk Wilms tumor, patients with poor histologic and molecular characteristics, bilateral lesions, and recurrent disease have a much lower survival rate and deserve more attentions.^4^ Moreover, approximately 24% of Wilms tumor survivors are at a higher risk of other diseases, including second primary cancers, infertility, and cardiac disease.^5^, ^6^ In the clinical, although Wilms tumor patients have a high cure rate, high‐risk patients still have a very poor prognosis. Therefore, we must fully understand the polymorphisms in Wilms tumor patients to find more effective, cheaper, and safer treatment. At the same time, understanding the genetic inheritance of Wilms tumor is also an important premise to improve the prognosis of patients.

Some causal gene mutations have been reported in Wilms tumor. For instance, individuals with *WT1* mutations have an enormous risk of Wilms tumor.^7^, ^8^ A study by Ciceri et al suggested that constitutional anomalies of *CHEK2* play an important role in the development of Wilms tumor.^9^ Moreover, a series of genetic alterations were identified associate with Wilms tumor susceptibility, including *WTX*,^10^ *CITED1*,^11^ *SIX1*,^12^ *SIX2*,^13^ *TP53*,^14^, ^15^ *HACE1*,^16^ *LIN28B*,^17^ and *KRAS*.^18^ Nevertheless, these genetic variations are not sufficient to explain the whole genetic pathogenesis involved in Wilms tumor.

MYCN encoded by the *MYCN* proto‐oncogene is a *MYC* gene family member. This protein plays an indispensable role in the processes of cell growth, proliferation, differentiation, and apoptosis.^19^, ^20^, ^21^ In recent years, several groups reported amplified *MYCN* gene in different human cancers, such as human neuroblastoma, small cell lung carcinomas, astrocytoma, and retinoblastoma.^22^, ^23^ In addition, *MYCN* copy number increased in 17.5% of basal cell carcinoma patients.^24^ Despite all these achievements, the implication of *MYCN* gene in Wilms tumor remains not fully clarified, especially the effects of potentially functional single nucleotide polymorphisms (SNPs). Thus, we conducted this study to identify the association between *MYCN* SNPs and Wilms tumor susceptibility.

## 2 MATERIALS AND METHODS

### 2.1 Study subjects

In this hospital‐based epidemiological study, we recruited 183 patients and 603 controls from Yuying Children's Hospital of Wenzhou Medical University and Guangzhou Women and Children's Medical Center. Age, gender, and clinical stage distributions of all participants are summarized in Table S1. All the guardians of participants were voluntary to donate blood samples and signed informed consent forms. All individuals were independently recruited. This study was permitted by the Institutional Review Board of both the centers.

### 2.2 SNP selection and genotyping

Four polymorphisms (rs57961569 G > A, rs9653226 T > C, rs13034994 A > G, and rs60226897 G > A) in the *MYCN* gene were selected in this study as we described previously.^25^ Specific criteria of SNPs selection and genotyping methods have been described in our previous study.^26^, ^27^, ^28^ All the above four polymorphisms are located at the binding sites of transcription factors, which have potential effects on the binding ability of transcription factors. In addition, they all have a minor allele frequency greater than 5% in the Chinese population.

### 2.3 Statistical analysis

Hardy‐Weinberg equilibrium (HWE) among controls was calculated using goodness‐of‐fit chi‐squared test. And the differences in demographic variables and SNPs frequencies between cases and controls were tested using two‐sided chi‐squared test. Adjusted odds ratios (ORs) and 95% confidence intervals (CIs) were adopted to appraise the association of *MYCN* gene SNPs with Wilms tumor risk. Difference between groups was deemed to be effective significant when *P* < .05. All of the statistical analyses were processed with the version 9.4 SAS (SAS Institute, Cary, NC).

## 3 RESULTS

### 3.1 Characteristics of study population

The characteristics of subjects from Wenzhou and Guangzhou are summarized in Table S1. All the participants were under the age of 15 years, and the mean age was 29.64 months (±25.71, range = 1‐144 months) for cases and 29.00 months (±24.00, range = 0.07‐156 months) for controls, respectively. The discrepancy in age and gender between the cases and controls was not statistically significant (*P* = .486 for age and *P* = .997 for gender). In the case group, 6.56% of the patients were in clinical stage I, 30.05% were in II, 31.69% were in III, 21.86% were in IV, and 9.84% were not available (NA).

### 3.2 Correlation of *MYCN* gene polymorphisms with Wilms tumor risk

The detailed results are presented in Table 1. None of the four selected polymorphisms showed obvious deviation from the HWE in the control groups (*P* = .582 for the rs57961569 G > A polymorphism, *P* = .719 for the rs9653226 T > C polymorphism, *P* = .581 for the rs13034994 A > G polymorphism, *P* = .377 for the rs60226897 G > A polymorphism). Overall, no significant association was found between *MYCN* gene polymorphisms and Wilms tumor risk. No significant results were observed for the combined effect of risk genotypes either.

**Table 1:** Association between *MYCN* gene polymorphisms and Wilms tumor susceptibility

Genotype	Cases (N = 183)	Controls (N = 603)	*P* a	Crude OR (95% CI)	*P*	Adjusted OR (95% CI)^b^	*P* b
rs57961569 G > A (HWE = 0.582)							
GG	90 (49.18)	259 (42.95)		1.00		1.00	
GA	74 (40.44)	277 (45.94)		0.77 (0.54‐1.09)	.142	0.77 (0.54‐1.09)	.140
AA	19 (10.38)	67 (11.11)		0.82 (0.47‐1.43)	.479	0.82 (0.47‐1.44)	.481
Additive			.325	0.85 (0.66‐1.10)	.215	0.85 (0.66‐1.10)	.215
Dominant	93 (50.82)	344 (57.05)	.138	0.78 (0.56‐1.08)	.138	0.78 (0.56‐1.08)	.137
Recessive	164 (89.62)	536 (88.89)	.782	0.93 (0.54‐1.59)	.782	0.93 (0.54‐1.59)	.787
rs9653226 T > C (HWE = 0.719)							
TT	59 (32.24)	201 (33.33)		1.00		1.00	
TC	89 (48.63)	298 (49.42)		1.02 (0.70‐1.48)	.928	1.01 (0.70‐1.48)	.944
CC	35 (19.13)	104 (17.25)		1.15 (0.71‐1.85)	.577	1.15 (0.71‐1.85)	.580
Additive			.840	1.06 (0.84‐1.35)	.613	1.06 (0.84‐1.35)	.618
Dominant	124 (67.76)	402 (66.67)	.783	1.05 (0.74‐1.50)	.783	1.05 (0.74‐1.49)	.796
Recessive	148 (80.87)	499 (82.75)	.560	1.14 (0.74‐1.74)	.560	1.14 (0.74‐1.74)	.556
rs13034994 A > G (HWE = 0.581)							
AA	104 (56.83)	350 (58.04)		1.00		1.00	
AG	67 (36.61)	222 (36.82)		1.02 (0.72‐1.44)	.931	1.02 (0.72‐1.45)	.916
GG	12 (6.56)	31 (5.14)		1.30 (0.65‐2.63)	.460	1.30 (0.65‐2.63)	.458
Additive			.759	1.08 (0.82‐1.41)	.603	1.08 (0.82‐1.42)	.592
Dominant	79 (43.17)	253 (41.96)	.771	1.05 (0.75‐1.47)	.771	1.06 (0.75‐1.48)	.756
Recessive	171 (93.44)	572 (94.86)	.461	1.30 (0.65‐2.58)	.462	1.30 (0.65‐2.58)	.462
rs60226897 G > A (HWE = 0.377)							
GG	92 (50.27)	293 (48.59)		1.00		1.00	
GA	69 (37.70)	248 (41.13)		0.89 (0.62‐1.26)	.504	0.89 (0.62‐1.27)	.508
AA	22 (12.02)	62 (10.28)		1.13 (0.66‐1.94)	.657	1.14 (0.66‐1.95)	.647
Additive			.642	1.00 (0.78‐1.28)	.992	1.00 (0.78‐1.28)	.981
Dominant	91 (49.73)	310 (51.41)	.690	0.94 (0.67‐1.30)	.690	0.94 (0.67‐1.30)	.697
Recessive	161 (87.98)	541 (89.72)	.505	1.19 (0.71‐2.00)	.505	1.20 (0.71‐2.01)	.496
Combined effect of risk genotypes							
0‐1	85 (46.45)	328 (54.39)		1.00		1.00	
2‐4	98 (53.55)	275 (45.61)	.059	1.38 (0.99‐1.92)	0.058	1.38 (0.99‐1.92)	.058

a χ^2^ test for genotype distributions between Wilms tumor patients and controls.

b Adjusted for age and gender.

Risk genotype was with rs57961569 GG, rs9653226 TC/CC, rs13034994 AG/GG, and rs60226897 AA.

### 3.3 Stratification analysis

Stratification analysis was performed according to age, sex, and clinical stages (Table 2). The rs57961569 GA/AA genotypes significantly decreased Wilms tumor susceptibility in the children older than 18 months (adjusted OR = 0.65, 95% CI = 0.42‐1.00, *P* = .050). In addition, children with 2‐4 risk genotypes have a greater risk of developing Wilms tumor than those with 0‐1 risk genotype (OR = 1.55, 95% CI = 1.001‐2.40, *P* = .0497). However, the association was not statistically significant after adjusting for age and gender, which turns out that age and gender were confounding factors.

**Table 2:** Stratification analysis of risk genotypes with tumor susceptibility

Variables	rs57961569 (cases/controls)		OR (95% CI)	*P*	AOR (95% CI)^a^	*P* a	Risk genotypes (cases/controls)		OR (95% CI)	*P*	AOR (95% CI)^a^	*P* a
	GG	GA/AA					0‐1	2‐4				
Age, month												
≤18	35/124	41/144	1.01 (0.61‐1.68)	.973	1.02 (0.61‐1.70)	.937	35/135	41/133	1.19 (0.71‐1.98)	.506	1.18 (0.71‐1.96)	.534
>18	55/135	52/200	**0.64 (0.41‐0.99)**	**.044**	**0.65 (0.42‐1.00)**	**.050**	50/193	57/142	**1.55 (1.001‐2.40)**	**.0497**	1.54 (0.99‐2.38)	.056
Gender												
Female	40/119	41/148	0.82 (0.50‐1.36)	.447	0.82 (0.50‐1.36)	.447	37/142	44/125	1.35 (0.82‐2.23)	.237	1.35 (0.82‐2.23)	.234
Male	50/140	52/196	0.74 (0.48‐1.16)	.190	0.74 (0.48‐1.16)	.188	48/186	54/150	1.40 (0.90‐2.18)	.142	1.40 (0.90‐2.18)	.139
Clinical stages												
I + II	34/259	33/344	0.73 (0.44‐1.21)	.224	0.74 (0.45‐1.23)	.248	30/328	37/275	1.47 (0.89‐2.44)	.136	1.44 (0.86‐2.39)	.163
III + IV	46/259	52/344	0.85 (0.56‐1.31)	.461	0.85 (0.55‐1.31)	.456	48/328	50/275	1.24 (0.81‐1.91)	.319	1.26 (0.82‐1.93)	.298

a Adjusted for age and gender, without the corresponding stratify factor. 1

### 3.4 Haplotype analysis

As shown in the Table 3, it is obvious that haplotype GCAA significantly increased Wilms tumor risk (adjusted OR = 2.40, 95% CI = 1.12‐5.14, *P* = .024) compared to reference haplotype GTAG.

**Table 3:** The frequency of inferred haplotypes of *MYCN* gene based on observed genotypes and their association with the risk of Wilms tumor

Haplotypes^a^	Cases (n = 366)	Controls (n = 1206)	Crude OR (95% CI)	*P*	Adjusted OR^b^ (95% CI)	*P* b
GTAG	91 (24.86)	343 (28.44)	1.00		1.00	
GTAA	5 (1.37)	13 (1.08)	1.45 (0.50‐4.17)	.491	1.47 (0.51‐4.23)	.477
GTGG	22 (6.01)	62 (5.14)	1.34 (0.78‐2.29)	.290	1.34 (0.78‐2.30)	.288
GTGA	2 (0.55)	2 (0.17)	3.77 (0.52‐27.12)	.188	3.85 (0.54‐27.77)	.181
GCAG	63 (17.21)	191 (15.84)	1.24 (0.86‐1.79)	.245	1.24 (0.86‐1.80)	.243
GCAA	12 (3.28)	19 (1.58)	2.38 (1.12‐5.08)	.025	2.40 (1.12‐5.14)	.024
GCGG	44 (12.02)	113 (9.45)	1.46 (0.96‐2.21)	.079	1.46 (0.96‐2.22)	.076
GCGA	4 (1.09)	17 (1.41)	0.89 (0.29‐2.70)	.833	0.89 (0.29‐2.72)	.843
ATAG	8 (2.19)	20 (1.66)	1.51 (0.64‐3.53)	.345	1.51 (0.64‐3.54)	.343
ATAA	33 (9.02)	116 (9.62)	1.07 (0.68‐1.68)	.761	1.08 (0.69‐1.69)	.750
ATGG	7 (1.91)	28 (2.32)	0.94 (0.40‐2.23)	.892	0.94 (0.40‐2.23)	.891
ATGA	20 (5.46)	80 (6.63)	0.94 (0.55‐1.62)	.830	0.95 (0.55‐1.63)	.853
ACAG	8 (2.19)	42 (3.48)	0.72 (0.33‐1.58)	.411	0.72 (0.33‐1.58)	.407
ACAA	46 (12.57)	158 (13.10)	1.10 (0.73‐1.64)	.650	1.10 (0.73‐1.64)	.654
ACGG	1 (0.27)	1 (0.08)	3.77 (0.23‐60.84)	.350	3.89 (0.24‐63.07)	.340

a The haplotypes' order was rs57961569, rs9653226, rs13034994, and rs60226897.

b Obtained in logistic regression models with adjustment for age and gender.

## 4 DISCUSSION

We performed the current study to investigate the association of *MYCN* gene polymorphisms with Wilms tumor risk. We found no significant association between four included polymorphisms of *MYCN* and Wilms tumor risk in 183 cases and 603 controls. However, stratification and haplotype analyses revealed the potential contribution of *MYCN* polymorphisms to Wilms tumor risk.

Several groups have explored the influence of *MYCN* gene on Wilms tumor. It is proved that the expression level of *MYCN* increased in Wilms tumor,^29^, ^30^, ^31^, ^32^ and *MYCN* gene can up‐regulate the expression of *CRABP‐II* in Wilms tumor patients to promote tumor progression.^33^ A study executed by Williams et al showed that *MYCN* gene contributed to the development of Wilms tumor through manifold mechanisms.^34^ In addition, the effect of *MYCN* gene polymorphisms on Wilms tumor may be related to RA signaling pathway.^35^ The contradiction of results may be caused by the small sample size and racial differences.

Moreover, a large number of studies have demonstrated that the *MYCN* gene is closely related to neuroblastoma.^36^, ^37^, ^38^, ^39^, ^40^, ^41^ A study by Zaatiti et al indicated that neuroblastoma IMR‐32 cell lines had significantly decreased proliferation ability after *MYCN* knockout.^42^ *MYCN* not only is related to the development of neuroblastoma, but also is one of the most powerful prognostic markers of neuroblastoma. We have delved the association between *MYCN* gene SNPs and neuroblastoma susceptibility before, and we found that SNP rs57961569 G > A was significantly associated with neuroblastoma risk.^25^ MYCN also regulated the regulatory circuits of genes involved in the progression of neuroblastoma through TFAP4.^43^ Moreover, a study by Liu et al found that MYCN was involved in the malignant characteristics of erythroleukemia by inhibiting the activation of p21.^44^ What's more, amplified *MYCN* gene was observed in rhabdomyosarcoma,^45^ pediatric T‐cell acute lymphoblastic leukemia,^46^ astrocytoma, and meningioma.^47^

Our study is the first investigation to explore the relationship of *MYCN* gene SNPs with Wilms tumor susceptibility. There are several flaws in our research. First, only four genotypes were included in our research. The association between other *MYCN* polymorphisms and Wilms tumor susceptibility should be further explored. Secondly, the relatively small sample size, because of the low incidence of Wilms tumors, led to limited statistical power. Additionally, the environmental factors or environment‐genes interaction were not considered in this study.

In conclusion, no significant association was found between *MYCN* gene polymorphisms and Wilms tumor risk in overall analysis, but stratification and haplotype analyses suggested that *MYCN* gene polymorphisms might be low penetrant variants in Wilms tumor susceptibility. Studies with larger sample size are need to verify our finding.

## CONFLICTS OF INTEREST

The authors declare that there are no conflicts of interest.

## Footnotes

1 Bold values indicate the 95% CI excluded 1 or *P* < .05.

## References

[1] Kaneko Y , Okita H , Haruta M , et al. A high incidence of WT1 abnormality in bilateral Wilms tumours in Japan, and the penetrance rates in children with WT1 germline mutation. Br J Cancer. 2015;112(6):1121‐1133. 25688735 10.1038/bjc.2015.13 PMC4366886

[2] Diets IJ , Hoyer J , Ekici AB , et al. TRIM28 haploinsufficiency predisposes to Wilms tumor. Int J Cancer. 2019;145(4):941‐951. 30694527 10.1002/ijc.32167

[3] Stokes CL , Stokes WA , Kalapurakal JA , et al. Timing of radiation therapy in pediatric Wilms tumor: a report from the national cancer database. Int J Radiat Oncol Biol Phys. 2018;101(2):453‐461. 29559286 10.1016/j.ijrobp.2018.01.110

[4] Dome JS , Graf N , Geller JI , et al. Advances in Wilms tumor treatment and biology: progress through international collaboration. J Clin Oncol. 2015;33(27):2999‐3007. 26304882 10.1200/JCO.2015.62.1888 PMC4567702

[5] Gratias EJ , Dome JS , Jennings LJ , et al. Association of chromosome 1q gain with inferior survival in favorable‐histology Wilms tumor: a report from the children's oncology group. J Clin Oncol. 2016;34(26):3189‐3194. 27400937 10.1200/JCO.2015.66.1140 PMC5012705

[6] Wong KF , Reulen RC , Winter DL , et al. Risk of adverse health and social outcomes up to 50 years after Wilms tumor: the British childhood cancer survivor study. J Clin Oncol. 2016;34(15):1772‐1779. 27022116 10.1200/JCO.2015.64.4344

[7] Little SE , Hanks SP , King‐Underwood L , et al. Frequency and heritability of WT1 mutations in nonsyndromic Wilms' tumor patients: a UK Children's Cancer Study Group Study. J Clin Oncol. 2004;22(20):4140‐4146. 15483024 10.1200/JCO.2004.02.136

[8] Kim MK , Mason JM , Li CM , et al. A pathologic link between Wilms tumor suppressor gene, WT1, and IFI16. Neoplasia. 2008;10(1):69‐78. 18231640 10.1593/neo.07869 PMC2213901

[9] Ciceri S , Gamba B , Corbetta P , et al. Genetic and epigenetic analyses guided by high resolution whole‐genome SNP array reveals a possible role of CHEK2 in Wilms tumour susceptibility. Oncotarget. 2018;9(75):34079‐34089. 30344923 10.18632/oncotarget.26123 PMC6183341

[10] Rivera MN , Kim WJ , Wells J , et al. The tumor suppressor WTX shuttles to the nucleus and modulates WT1 activity. Proc Natl Acad Sci USA. 2009;106(20):8338‐8343. 19416806 10.1073/pnas.0811349106 PMC2677091

[11] Murphy AJ , Pierce J , de Caestecker C , et al. CITED1 confers stemness to Wilms tumor and enhances tumorigenic responses when enriched in the nucleus. Oncotarget. 2014;5(2):386‐402. 24481423 10.18632/oncotarget.1566 PMC3964215

[12] Sehic D , Ciornei CD , Gisselsson D . Evaluation of CITED1, SIX1, and CD56 protein expression for identification of blastemal elements in Wilms tumor. Am J Clin Pathol. 2014;141(6):828‐833. 24838327 10.1309/AJCPQ8Q0YTHSYZTX

[13] Walz AL , Ooms A , Gadd S , et al. Recurrent DGCR13, DROSHA, and SIX homeodomain mutations in favorable histology Wilms tumors. Cancer Cell. 2015;27(2):286‐297. 25670082 10.1016/j.ccell.2015.01.003 PMC4800737

[14] Liu P , Zhuo Z , Li W , et al. TP53 rs1042522 C>G polymorphism and Wilms tumor susceptibility in Chinese children: a four‐center case‐control study. Biosci Rep. 2019;39(1):BSR20181891. 30610160 10.1042/BSR20181891 PMC6340947

[15] Treger TD , Chagtai T , Butcher R , et al. Somatic TP53 mutations are detectable in circulating tumor DNA from children with anaplastic Wilms tumors. Transl Oncol. 2018;11(6):1301‐1306. 30172241 10.1016/j.tranon.2018.08.006 PMC6121832

[16] Jia W , Deng Z , Zhu J , et al. Association between HACE1 gene polymorphisms and Wilms' tumor risk in a Chinese population. Cancer Invest. 2017;35(10):633‐638. 29243987 10.1080/07357907.2017.1405016

[17] Fu W , Liu GC , Zhao Z , et al. The correlation between LIN28B gene potentially functional variants and Wilms tumor susceptibility in Chinese children. J Clin Lab Anal. 2018;32(1):e22200. 10.1002/jcla.22200 PMC6817198 28301057

[18] Fu W , Zhuo Z , Hua RX , et al. Association of KRAS and NRAS gene polymorphisms with Wilms tumor risk: a four‐center case‐control study. Aging (Albany NY). 2019;11(5):1551‐1563. 30860980 10.18632/aging.101855 PMC6428095

[19] Somasundaram DB , Aravindan S , Yu Z , et al. Droplet digital PCR as an alternative to FISH for MYCN amplification detection in human neuroblastoma FFPE samples. BMC Cancer. 2019;19(1):106. 30691436 10.1186/s12885-019-5306-0 PMC6348625

[20] Okubo T , Knoepfler PS , Eisenman RN , Hogan BL . Nmyc plays an essential role during lung development as a dosage‐sensitive regulator of progenitor cell proliferation and differentiation. Development. 2005;132(6):1363‐1374. 15716345 10.1242/dev.01678

[21] Yoda H , Inoue T , Shinozaki Y , et al. Direct targeting of MYCN gene amplification by site‐specific DNA alkylation in neuroblastoma. Cancer Res. 2019;79(4):830‐840. 30584073 10.1158/0008-5472.CAN-18-1198

[22] Breit S , Schwab M . Suppression of MYC by high expression of NMYC in human neuroblastoma cells. J Neurosci Res. 1989;24(1):21‐28. 2810395 10.1002/jnr.490240105

[23] Ikegaki N , Bukovsky J , Kennett RH . Identification and characterization of the NMYC gene product in human neuroblastoma cells by monoclonal antibodies with defined specificities. Proc Natl Acad Sci USA. 1986;83(16):5929‐5933. 2426708 10.1073/pnas.83.16.5929 PMC386410

[24] Freier K , Flechtenmacher C , Devens F , et al. Recurrent NMYC copy number gain and high protein expression in basal cell carcinoma. Oncol Rep. 2006;15(5):1141‐1145. 16596176

[25] Zhou H , Zhuo Z , Chen S , et al. Polymorphisms in MYCN gene and neuroblastoma risk in Chinese children: a 3‐center case‐control study. Cancer Manag Res. 2018;10:1807‐1816. 29997440 10.2147/CMAR.S168515 PMC6033082

[26] He J , Qiu LX , Wang MY , et al. Polymorphisms in the XPG gene and risk of gastric cancer in Chinese populations. Hum Genet. 2012;131(7):1235‐1244. 22371296 10.1007/s00439-012-1152-8

[27] He J , Wang F , Zhu J , et al. Association of potentially functional variants in the XPG gene with neuroblastoma risk in a Chinese population. J Cell Mol Med. 2016;20(8):1481‐1490. 27019310 10.1111/jcmm.12836 PMC4956948

[28] Wang J , Zhuo Z , Chen M , et al. RAN/RANBP2 polymorphisms and neuroblastoma risk in Chinese children: a three‐center case‐control study. Aging (Albany NY). 2018;10(4):808‐818. 29706609 10.18632/aging.101429 PMC5940104

[29] Williams RD , Al‐Saadi R , Natrajan R , et al. Molecular profiling reveals frequent gain of MYCN and anaplasia‐specific loss of 4q and 14q in Wilms tumor. Genes Chromosomes Cancer. 2011;50(12):982‐995. 21882282 10.1002/gcc.20907

[30] Williams RD , Al‐Saadi R , Chagtai T , et al. Subtype‐specific FBXW7 mutation and MYCN copy number gain in Wilms' tumor. Clin Cancer Res. 2010;16(7):2036‐2045. 20332316 10.1158/1078-0432.CCR-09-2890 PMC5122447

[31] Noguera R , Villamon E , Berbegall A , et al. Gain of MYCN region in a Wilms tumor‐derived xenotransplanted cell line. Diagn Mol Pathol. 2010;19(1):33‐39. 20186010 10.1097/PDM.0b013e3181b11a9d

[32] Micale MA , Bt E , Macknis JK , Harper CE , Aughton DJ . Constitutional 560.49 kb chromosome 2p24.3 duplication including the MYCN gene identified by SNP chromosome microarray analysis in a child with multiple congenital anomalies and bilateral Wilms tumor. Eur J Med Genet. 2016;59(12):618‐623. 27794475 10.1016/j.ejmg.2016.10.010

[33] Gupta A , Kessler P , Rawwas J , Williams BR . Regulation of CRABP‐II expression by MycN in Wilms tumor. Exp Cell Res. 2008;314(20):3663‐3668. 18955045 10.1016/j.yexcr.2008.09.029

[34] Williams RD , Chagtai T , Alcaide‐German M , et al. Multiple mechanisms of MYCN dysregulation in Wilms tumour. Oncotarget. 2015;6(9):7232‐7243. 25749049 10.18632/oncotarget.3377 PMC4466681

[35] Wegert J , Bausenwein S , Kneitz S , et al. Retinoic acid pathway activity in Wilms tumors and characterization of biological responses in vitro. Mol Cancer. 2011;10:136. 22067876 10.1186/1476-4598-10-136 PMC3239322

[36] Campbell K , Gastier‐Foster JM , Mann M , et al. Association of MYCN copy number with clinical features, tumor biology, and outcomes in neuroblastoma: A report from the Children's Oncology Group. Cancer. 2017;123(21):4224‐4235. 28696504 10.1002/cncr.30873 PMC5650521

[37] Kerosuo L , Neppala P , Hsin J , et al. Enhanced expression of MycN/CIP2A drives neural crest toward a neural stem cell‐like fate: Implications for priming of neuroblastoma. Proc Natl Acad Sci USA. 2018;115(31):E7351‐E7360. 30021854 10.1073/pnas.1800039115 PMC6077707

[38] Dzieran J , Rodriguez Garcia A , Westermark UK , et al. MYCN‐amplified neuroblastoma maintains an aggressive and undifferentiated phenotype by deregulation of estrogen and NGF signaling. Proc Natl Acad Sci USA. 2018;115(6):E1229‐E1238. 29374092 10.1073/pnas.1710901115 PMC5819392

[39] Zhong ZY , Shi BJ , Zhou H , Wang WB . CD133 expression and MYCN amplification induce chemoresistance and reduce average survival time in pediatric neuroblastoma. J Int Med Res. 2018;46(3):1209‐1220. 29322842 10.1177/0300060517732256 PMC5972240

[40] Berbegall AP , Bogen D , Potschger U , et al. Heterogeneous MYCN amplification in neuroblastoma: a SIOP Europe Neuroblastoma Study. Br J Cancer. 2018;118(11):1502‐1512. 29755120 10.1038/s41416-018-0098-6 PMC5988829

[41] Zhang P , Wu X , Basu M , et al. MYCN amplification is associated with repressed cellular immunity in neuroblastoma: an in silico immunological analysis of TARGET database. Front Immunol. 2017;8:1473. 29163537 10.3389/fimmu.2017.01473 PMC5675839

[42] Zaatiti H , Abdallah J , Nasr Z , Khazen G , Sandler A , Abou‐Antoun TJ . Tumorigenic proteins upregulated in the MYCN‐amplified IMR‐32 human neuroblastoma cells promote proliferation and migration. Int J Oncol. 2018;52(3):787‐803. 29328367 10.3892/ijo.2018.4236 PMC5807036

[43] Xue C , Yu DM , Gherardi S , et al. MYCN promotes neuroblastoma malignancy by establishing a regulatory circuit with transcription factor AP4. Oncotarget. 2016;7(34):54937‐54951. 27448979 10.18632/oncotarget.10709 PMC5342392

[44] Liu L , Xu F , Chang CK , et al. MYCN contributes to the malignant characteristics of erythroleukemia through EZH2‐mediated epigenetic repression of p21. Cell Death Dis. 2017;8(10):e3126. 29022893 10.1038/cddis.2017.526 PMC5682688

[45] O'Brien EM , Selfe JL , Martins AS , Walters ZS , Shipley JM . The long non‐coding RNA MYCNOS‐01 regulates MYCN protein levels and affects growth of MYCN‐amplified rhabdomyosarcoma and neuroblastoma cells. BMC Cancer. 2018;18(1):217. 29466962 10.1186/s12885-018-4129-8 PMC5822637

[46] Astolfi A , Vendemini F , Urbini M , et al. MYCN is a novel oncogenic target in pediatric T‐cell acute lymphoblastic leukemia. Oncotarget. 2014;5(1):120‐130. 24334727 10.18632/oncotarget.1337 PMC3960194

[47] Estiar MA , Javan F , Zekri A , Mehrazin M , Mehdipour P . Prognostic significance of MYCN gene amplification and protein expression in primary brain tumors: Astrocytoma and meningioma. Cancer Biomark. 2017;19(3):341‐351. 28453467 10.3233/CBM-160546 PMC13020733

