# Supplementary material for: MYCN gene polymorphisms and Wilms tumor susceptibility in Chinese children
Source: J Clin Lab Anal. 2019 Jul 25;33(9):e22988. doi: 10.1002/jcla.22988 (PMC7938399; doi:10.1002/jcla.22988)
Supplement: Supplementary file 1 [file JCLA-33-e22988-s001.doc]

| **Supplemental Table 1**. Frequency distribution of selected variables for Wilms tumor patients and cancer-free controls | | | | | |
| --- | --- | --- | --- | --- | --- |
| Variables | Cases (n=183) | | Controls (n=603) | | *P ^a^* |
|  | No. | % | No. | % |  |
| Age range, month | 1-144 | | 0.07-156 | | 0.486 |
| Mean ± SD | 29.64 ± 25.71 | | 29.00 ± 24.00 | |  |
| ≤18 | 76 | 41.53 | 268 | 44.44 |  |
| >18 | 107 | 58.47 | 335 | 55.56 |  |
| Gender |  |  |  |  | 0.997 |
| Female | 81 | 44.26 | 267 | 44.28 |  |
| Male | 102 | 55.74 | 336 | 55.72 |  |
| Clinical stages |  |  |  |  |  |
| I | 12 | 6.56 |  |  |  |
| II | 55 | 30.05 |  |  |  |
| III | 58 | 31.69 |  |  |  |
| IV | 40 | 21.86 |  |  |  |
| NA | 18 | 9.84 |  |  |  |
| ^a^ Two-sided χ^2^ test for distributions between Wilms tumor patients and cancer-free controls. | | | | | |
